# FPGA Correlator for Applications in Embedded Smart Devices

**DOI:** 10.3390/bios12040236

**Published:** 2022-04-12

**Authors:** Christopher H. Moore, Wei Lin

**Affiliations:** Department of Biomedical Engineering, Stony Brook University, Stony Brook, NY 11794, USA; christopher.h.moore@stonybrook.edu

**Keywords:** FPGA correlator, diffuse correlation spectroscopy, dynamic light scattering, fluorescence correlation spectroscopy, device-on-chip

## Abstract

Correlation has a variety of applications that require signal processing. However, it is computationally intensive, and software correlators require high-performance processors for real-time data analysis. This is a challenge for embedded devices because of the limitation of computing resources. Hardware correlators that use Field Programmable Gate Array (FPGA) technology can significantly boost computational power and bridge the gap between the need for high-performance computing and the limited processing power available in embedded devices. This paper presents a detailed FPGA-based correlator design at the register level along with the open-source Very High-Speed Integrated Circuit Hardware Description Language (VHDL) code. It includes base modules for linear and multi-tau correlators of varying sizes. Every module implements a simple and unified data interface for easy integration with standard and publicly available FPGA modules. Eighty-lag linear and multi-tau correlators were built for validation of the design. Three input data sets—constant signal, pulse signal, and sine signal—were used to test the accuracy of the correlators. The results from the FPGA correlators were compared against the outputs of equivalent software correlators and validated with the corresponding theoretical values. The FPGA correlators returned results identical to those from the software references for all tested data sets and were proven to be equivalent to their software counterparts. Their computation speed is at least 85,000 times faster than the software correlators running on a Xilinx MicroBlaze processor. The FPGA correlator can be easily implemented, especially on System on a Chip (SoC) integrated circuits that have processor cores and FPGA fabric. It is the ideal component for device-on-chip solutions in biosensing.

## 1. Introduction

Correlation is a signal processing tool that can find the similarity between two signals. Autocorrelation is a special type of correlation where the two signals are from the same source. Correlation can determine signal periodicity [1] and detect signal delays [2]. It has broad applications in radar [3,4], image processing [5,6], biosignal analysis [7,8], and machine learning [9]. Autocorrelation is also widely used in optical biosensing applications such as Diffuse Correlation Spectroscopy (DCS) [10], Dynamic Light Scattering (DLS) [11], and Fluorescence Correlation Spectroscopy (FCS) [12,13], where the correlation of the detected photon counts is the key process in the data analysis. Since the scale of the lag times in those applications could have a dynamic range from nanoseconds to seconds, a multi-tau correlation scheme is used to generate a quasi-logarithmic scale that covers a wide range of the lag times [14].

Correlation is often computationally intensive, especially when data size is large. Thus, it is a challenge to use software approaches for the computation of the correlation in real-time data analysis when computing resources are limited. Hardware correlators have been developed using field-programmable gate array technology to address the need. For example, multi-channel FPGA cross-correlators were used in aperture synthesis imaging systems [15,16,17]. The FPGA correlator alleviated the computational load in real-time ultrasound sensor systems using the complementary ultrasound pulse sequences for the enhancement of the signal-to-noise ratio [18].

Most studies on FPGA correlators provided only high-level functional diagrams with little details of the actual design. This makes it difficult for interested researchers to replicate the work for their research projects. The papers on multi-tau FPGA correlators had more exposure of the internal design compared with other reports of FPGA correlators. Islambek et al. published the design of a real-time multi-tau autocorrelator for application in dynamic light scattering [19]. It implemented a finite state machine to control the operation of the correlator and used memory blocks for data storage. The correlator has submodules working at a series of frequencies. When the unit lag time of the correlation is doubled every few lags according to the multi-tau scheme, the frequency of the corresponding correlator module is reduced by half. However, the relationship between the decreasing frequencies and the multi-tau scheme was not clearly discussed. Similarly, Liu et al. also presented a simpler design of a multi-tau correlator using the cascaded connection of linear correlator modules without the need for the state machine and memory blocks [20]. Buchholz et al. reported a multi-channel multi-tau FPGA correlator [21] with a clear illustration of the implementation of a multi-tau scheme in hardware. Those designs have the following limitations: First, they are not modular designs, so they cannot be readily integrated into other applications. They are for specific projects and are not fit for general-purpose use. Second, they did not show scalability so that the size of the correlator, i.e., the number of output correlation values from the correlator, can be adjusted easily to accommodate the needs of actual applications. Third, there was no standard data interface in those designs that could integrate the correlator with standard FPGA IPs for data transfer and output. Lastly, no open-source code was provided for researchers to reproduce their correlators.

This paper presents the design of an FPGA correlator at the register level for general-purpose use. It follows the rules of modular design and produces basic FPGA modules as the building blocks of an FPGA correlator. It has a simple and unified data interface that matches standard FPGA IPs [22] for easy integration. It has the scalability to build correlators of any number of correlation time lags without any additional control circuits. It can be used to form linear correlators and multi-tau correlators for various applications. The objective of the paper is to validate the accuracy and high performance of the FPGA correlator.

Sensors are the front-end component of a measurement and control system. As technologies evolve, they are not just the devices to convert physical quantities into outputs that can be measured electronically. Additional data analysis features can turn sensors into smart devices. Microcontrollers are the driving force in smart devices. However, their computational resources are often limited compared with high-end processors. Hardware accelerators are the solution to alleviate this bottleneck. When combined with FPGA technology, microcontrollers can significantly enhance their data processing capability by moving the computational load from software to hardware. This can give microcontroller-based smart devices the computational power of a high-performance computer while maintaining small footprints for portable or wearable applications. The FPGA correlator is one of the solutions to boost the computational power of smart devices that need on-board real-time correlation analysis.

## 2. Methods

### 2.1. Discrete Correlation

For two discrete time series, *f* and *g*, with the same number of data points, the computation of the normalized cross-correlation function c(n) is defined by Equation (1), where N is the total number of data points in *f* or *g*, and n is the time lag or delay. When *f* and *g* are the same series, it becomes an autocorrelation.

(1) $$c\left(n\right)=\frac{1}{N-n}\overset{N-n}{\underset{i=1}{\sum }}f\left(i\right)g\left(i-n\right)$$

The multi-tau correlation scheme was devised for when the calculation of the correlation across a large range of lag times is needed. It increases the time of the unit lag in the correlation as the lag grows to generate a quasi-logarithmic time scale of the correlation lag. It is equivalent to downsampling the data. It has applications in optics where the lag of the autocorrelation of photon counts over decades of time scale is required. Our FPGA modules are scalable and can be used to build linear and multi-tau correlators easily to compute the correlation defined in Equation (1).

### 2.2. FPGA Correlator Design

The basic operation of the correlator is the multiplication–accumulation operation where two input values are multiplied and accumulated over the entire data series. The Digital Signal Processing (DSP) slices on the FPGA are ideal for implementing this operation [23]. For example, the Artix-7 series from Xilinx has DSP slices containing 25 × 18 two’s complement multipliers and 48-bit accumulators, which can be easily configured to perform the multiply–accumulate task of the correlation. Using the DSP slices reduces the utilization of FPGA fabric logic and allows for this operation to be performed efficiently.

The FPGA correlator design adopts the following rules for easy integration: First, the data inputs and outputs accompany active high valid signals. The input data are valid when the New Data (ND) signal is active high, while the output data are valid when the ready (Rdy) signal is active high. Second, the basic building block is the Single-Lag Correlator (SLC) module. The linear and multi-tau correlators built from this module have the same port configuration as the SLC module, so they can be integrated without additional support circuits.

#### 2.2.1. Single-Lag Correlator Module

**Figure 1:**
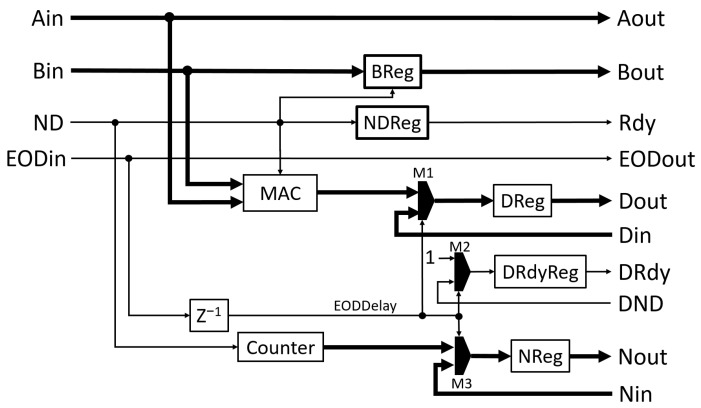
Functional block diagram of the SLC module that calculates the correlation value of single lag.

Figure 1 shows the functional diagram of the SLC module and Table 1 is the description of the inputs and outputs. Ain and Bin are the inputs of two data series. The data must be valid in the same clock when the ND signal is active high. An End-of-data (EOD) signal, EODin, will be active high with the ND signal to indicate the last data input. It also triggers the output of the multiplication accumulator (MAC) for the following normalization of the correlation values.

When ND is high, Ain and Bin are multiplied and added to a 32-bit accumulation register inside the MAC, which uses one DSP slice and has a latency of one clock cycle. At the same time, the counter module counts the pulses from ND so that the counter’s value is the number of data points used in the correlation. The registers (32-bit DReg, 16-bit NReg, and 1-bit DRdyReg), the inputs (Din, Nin, and DND), and the outputs (Dout, Nout, and DRdy) form data output shift registers. After a one-clock delay of the valid EODin signal, i.e., the EODDelay signal, the MAC places the value stored in the accumulation register into the DReg register. This is the value of the summation term in Equation (1), referred to as the raw correlation value. At the same time, the counter places its value into the register NReg. It is the item of N−n in Equation (1). The DRdy register is set high, indicating that the data in DReg and NReg are valid. Those operations are accomplished through the multiplexers M1, M2, and M3, which are controlled by the EODDelay signal. The values in those registers are output through the output terminals (Dout, Nout, and DRdy) at the next FPGA clock cycle. This one-clock delay (EODDelay) is derived from EODin through the EODReg register. The EODDelay signal also synchronously resets the counter and registers in the module for the next correlation computation. Thus, the same registers in the cascaded correlators can form a series of shift registers for the output of correlation values, which is illustrated in the following eight-lag correlator module (8-LC). When the EODin signal is not active, the registers will be updated with the inputs from Din, Nin, and DND. Therefore, setting Din and Nin as zero and DND as low can reset the registers at the next clock cycle. That is usually done to the last module in the cascade.

**Figure 2:**
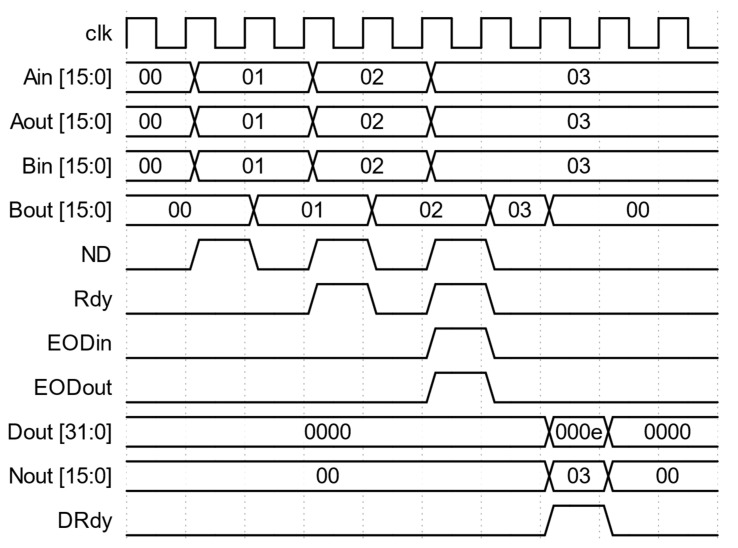
Waveforms of inputs and outputs for the SLC module. All values shown are in hexadecimal. The validity of the data is flagged by the corresponding active high valid signal.

Additional logic is implemented to make the SLC module reusable in a cascade connection to form a multi-lag correlator. The module has Aout and Bout outputs that can be used as Ain and Bin inputs, respectively, of the following correlator module. Internally, Aout is the direct pass-through of Ain. Bin is the input for the BReg register, which places its output on Bout. Additional delay based on the ND signal is introduced in BReg so that the data in the register are delayed by one ND pulse. Thus, the data at Bout are synchronized with the next data in Aout. This is the mechanism to generate the single lag needed for the following correlation calculation. The Rdy output is the signal to indicate the validity of the Aout and Bout data signals and can be connected to the following module’s ND input. Rdy will be active high with every ND pulse, excluding the first as Bin needs one ND pulse to propagate to Bout. This is accomplished by the logic inside NDReg. Like Aout, EODout is the direct pass-through of EODin. Figure 2 is the waveform diagram from the simulation of the SLC module. The inputs for Ain and Bin are the same data series, e.g., 1, 2, and 3. The Rdy signal is synchronized with the ND signal except for the first ND pulse. Bout is delayed by one ND pulse so that the values (1 and 2) of Bout are aligned with the values (2 and 3) of Aout, respectively, when Rdy is active high. The EODin and EODout signals indicate the last data pair and are synchronized with the ND signal. The raw correlation value of the data series at Ain and Bin is 14 (1 × 1 + 2 × 2 + 3 × 3) or 0x000E in hexadecimal, and the number of data points in the correlation is 3. They are available at Dout and Nout when DRdy is active high.

#### 2.2.2. Eight-Lag Correlator Module

**Figure 3:**
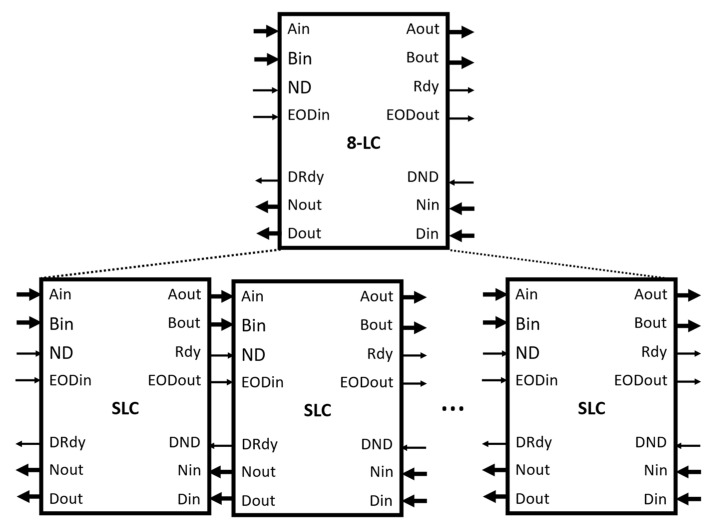
Creation of an 8-LC module from the SLC module. It has the same inputs and outputs as the SLC module.

A cascaded connection of multiple SLC modules allows for the direct formation of a linear correlator module of multiple values. For example, 8-LC is formed by chaining eight SLC modules together with the connections shown in Figure 3. The outputs of Aout, Bout, Rdy, and EODout of the previous SLC module are connected to inputs of Ain, Bin, ND, and EODin of the current SLC module. The outputs of Dout, Nout, and DRdy of the current SLC module are connected to the inputs of Din, Nin, and DND of the previous SLC module. That effectively creates three shift register chains using the registers of DReg, NReg, and DRdyReg in the eight SLC modules. When the EODin signal arrives, the values in the accumulators, the count values, and their valid signals are copied to their respective registers. The data in those registers are shifted out from the first module in series. For the last SLC module, its inputs of Din, Nin, and DND are the inputs of Din, Nin, and DND of the 8-LC module. Its output of Aout and Bout are the outputs of Aout and Bout of the 8-LC module.

**Figure 4:**
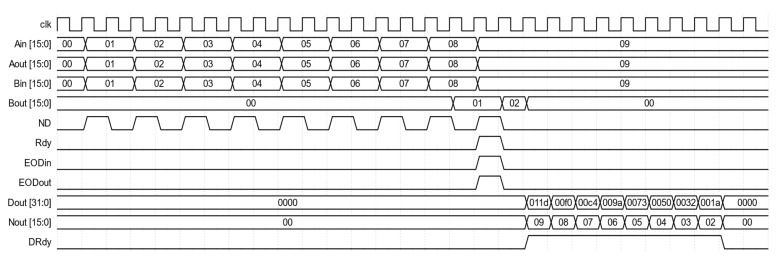
Waveforms of inputs and outputs for the 8-LC module. All values shown are in hexadecimal and are valid when their corresponding validation signals are active high.

The 8-LC module has the same input and output interfaces as the SLC module defined in Table 1. At each SLC module, the data at Bin have a unit delay compared to the data at Bin of the previous module. Therefore, the output at the Bout of the 8-LC module has eight unit delays. The 8-LC module is the building block of a correlator with a higher number of correlation lags. It is also the building block of the following multi-tau correlator. Figure 4 is the waveform diagram from the simulation of the 8-LC module. The input data consist of nine values at Ain and Bin, which are 1, 2, 3, 4, 5, 6, 7, 8, and 9. Bout is delayed by eight ND pulses and its only valid output, indicated by the Rdy signal, is the value 1, and it is aligned with the value 9 at Aout when the Rdy signal is active high. Dout outputs eight raw correlation values of the eight time lags after the EODin signal is active. They are the raw correlation values defined in Equation (1) with lags from 0 to 7, i.e., 285 (0x011d), 240 (0x00f0), 196 (0x00c4), 154 (0x009a), 115 (0x0073), 80 (0x0050), 50 (0x0032), and 26 (0x001a). Nout outputs the corresponding numbers of values used in the computation of the eight raw correlation values, which are 9, 8, 7, 6, 5, 4, 3, and 2. Their outputs are validated by the active high DRdy signal.

#### 2.2.3. Multi-Tau Correlator

One of the approaches in multi-tau correlation is to double the unit lag for every few correlation lags to create the quasi-logarithmic scales. The number of photons is counted within a preset time window, and the photon counts are the time series data for the correlation. The combination of the two consecutive photon-counting windows effectively doubles the photon sampling window and the unit lag of the correlation and creates the logarithmic time scale.

**Figure 5:**
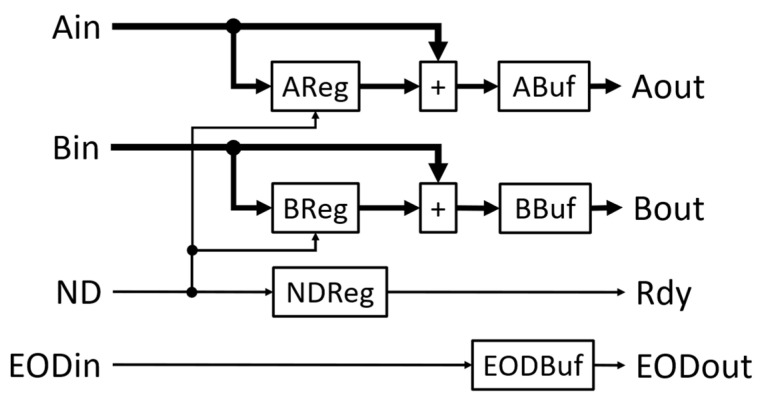
Functional block diagram of the combiner module. Reset signal (not shown in the figure) clears all registers in the module.

The mechanism of the multi-tau correlator in our design is to double the data sampling window after a fixed number of correlation lags. This can be accomplished by combining the data of two consecutive data samples in the data stream. For example, if the incoming data set is [1, 2, 3, 4…], the combined data set will be [3, 7…]. This effectively halves the number of data samples and doubles the signal magnitude. It also quadruples the accumulated value in the MAC, which will be addressed by the following scaling module. This combiner module, shown in Figure 5, has two major inputs and outputs for this module and their respective data valid signals. They are the 16-bit data inputs, Ain and Bin, with the data valid signal ND and the EODin signal, and the 16-bit data outputs, Aout and Bout, with the data valid signal Rdy and the EODout signal. This module is implemented by computing the sum of two consecutive values, but only asserting Rdy for every other valid ND signal. Internally, the AReg and BReg registers store the last valid values of Ain and Bin. The sums between AReg and Ain and between BReg and Bin are then calculated, stored in ABuf and BBuf, and output on Aout and Bout, respectively. Rdy is the output of NDReg, which toggles itself for each pulse of ND. The EODBuf buffer is used to synchronize EODout with the outputs of Aout, Bout, and Rdy as they are also buffered.

**Figure 6:**
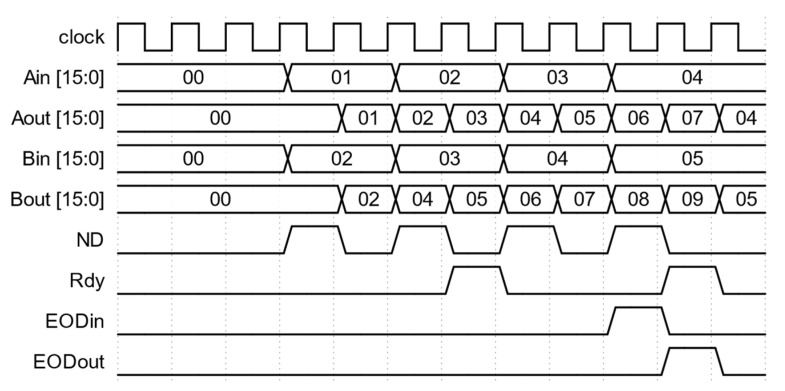
Waveforms of inputs and outputs for the combiner module. The Aout and Bout combine two consecutive values from Ain and Bin. The Rdy signal is active high every two valid ND pulses.

Figure 6 is the waveform diagram from the simulation of the combiner module. The inputs are 1, 2, 3, and 4 for Ain and 2, 3, 4, and 5 for Bin when ND is active high. Aout and Bout hold the values of the sum of Ain and Bin values at the current and previous clocks. However, only the values when the Rdy signal is active high are valid. They are 3 and 7 for Aout and 5 and 9 for Bout. Rdy is active with every other valid ND signal. The shift of Rdy and EODout signals by one FPGA clock compared to their counterpart ND and EODin signals is generated by the buffers in the module so that the output data and their corresponding valid signals are synchronized. This does not affect the operation of the following modules.

#### 2.2.4. Normalization of the Correlation Values

**Figure 7:**
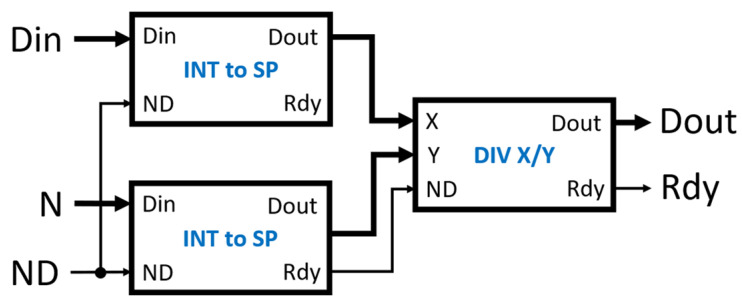
The diagram of the normalizer module. The INT to SP modules convert the integer inputs Din and N to single-precision floating-point numbers. The Rdy from either INT to SP module is used as the ND input of the division module (DIV X/Y) because both Rdy signals are already synchronized.

The normalization of the correlation values is the division of the raw correlation values from Dout by the number of samples used in the accumulation from Nout. Both values are synchronized and validated by DRdy. To preserve precision in this division operation, both integer outputs are converted into floating-point representations. A single-precision representation [24] was chosen because it provides sufficient precision while using a reasonable amount of the FPGA fabric resources. Xilinx’s floating-point IP [21] was used to convert the integers to single-precision floating-point numbers and perform the division operation. The IP was configured to use the minimum latency (12 FPGA clocks) that still met timing requirements. Figure 7 is the block diagram of the normalizer module linear correlator.

**Figure 8:**
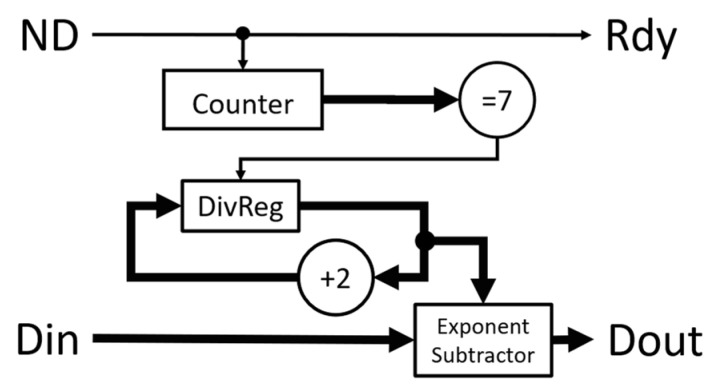
Functional block diagram of the scaler module. The input Din is a single-precision correlation value from the normalizer module. Dout is the final correlation value. Reset signal (not shown in the figure) clears all registers in the module.

As the combiner modules essentially double the input data in the multi-tau correlator, it is necessary to scale down the output data based on the linear correlator stage where the data come from. The output data need to be scaled down by a factor of 4^N^, where N is the number of combiner stages that the input data went through to generate that output. In our multi-tau correlator, there is no scaling necessary for the first 16 correlation values. For the next eight correlation values, the outputs need to be scaled down by a factor of 4. The scale factor will be quadrupled every eight lags thereafter. The scaling module takes advantage of the format of an IEEE 754 single-precision floating-point value, which breaks up the binary of the single-precision floating-point value into three parts: a sign bit, an 8-bit exponent, and a 23-bit fraction. The value represented by these parts is given by the expression ${\left(-1\right)}^{s}\times 2^{E-127}\times \left(1\times f_{1}f_{2}\ldots f_{23}\right)$ where s is the sign, E is the exponent, and $f_{i}$ are the fraction bits. Since the divisor is an exponent of 2, the division can be done by subtracting the value 2 from the exponent directly. This avoids the need for another floating-point IP for the division. The scaler module, shown in Figure 8, has two inputs, the single-precision floating-point data Din and the signal ND indicating when Din is valid. A 3-bit counter is used to count the ND pulses so that the value can be scaled down appropriately. With the exception of the first round of counts, each time the counter reaches 7, it increments the value to subtract from the exponent of the floating-point number by 2 and stores it in the DivReg register. This achieves division by 1 for the first 16 values and division by an increasing exponent of 4 for each subsequent group of 8, which cancels the quadrupling effect of the combiner modules. This module generates the final normalized 32-bit single-precision floating-point output values for the multi-tau correlation. This scaler is appended to the output of the previously described normalizer module when a multi-tau correlator is being made. The module has the latency of one FPGA clock.

### 2.3. Test Bench Setup

**Figure 9:**
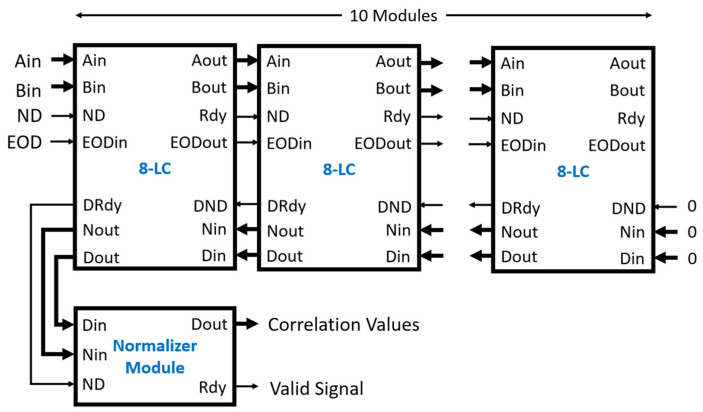
Linear correlator of 80 correlation values output. Input data are supplied to Ain and Bin with the control signals ND and EOD. The output of the correlator is from the Dout of the normalizer module. The Din and Nin of the last 8-LC module are set to zero and the DND is set to low.

An 80-lag linear correlator and an 80-lag multi-tau correlator were constructed for testing. Figure 9 shows the linear correlator when ten 8-LC modules were used. The connection of those modules is straightforward. For the linear correlator, the input data were connected to the Ain and Bin of the leftmost 8-LC module with the respective ND and EOD signals. The DND, Nin, and Din of the rightmost 8-LC module were connected to logic low and zero values, respectively. The outputs of the raw correlation values and the number of data points in the correlation are shifted out at Dout and Nout with the corresponding valid signal DRdy at the leftmost 8-LC module. These outputs act as inputs to the normalizer module. The outputs of the normalizer module, Dout, are the correlation values in the single-precision floating-point format validated by the active high Rdy output.

**Figure 10:**
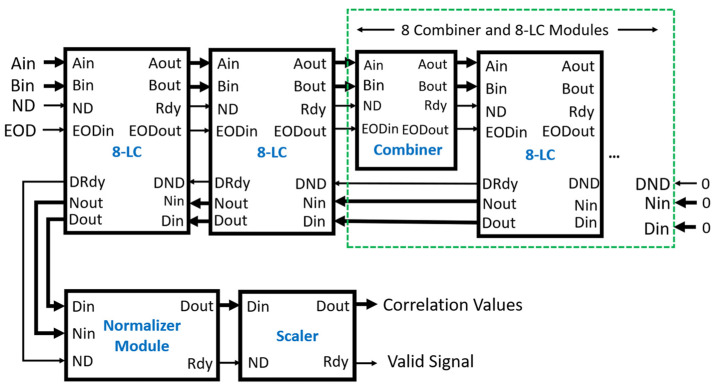
Multi-tau correlator of 80 correlation values output. Input data are supplied to Ain and Bin with the control signals ND and EOD. The output of the correlator is from the Dout of the scaler module. The Din and Nin of the last 8-LC module are set to zero and the DND is set to low.

The multi-tau correlator has the additional combiner and scaler modules, as shown in Figure 10. The combiner modules are inserted before each of the 8-LC modules, starting at the third one in the chain. The scaler module is added after the normalizer module, where the Dout and Rdy of the normalizer module are connected to the Din and ND of the scaler module, respectively.

A Digilent Arty A7 FPGA development board (Digilent Inc., Pullman, WA 99163, USA) was used to test the correlators. It is equipped with a Xilinx Artix-7 FPGA (XC7A100T) and 256 MB DDR3L memory. Two test benches were built using a Xilinx MicroBlaze soft microprocessor, one with the linear correlator and the other with the multi-tau correlator. The microprocessor was configured as a 32-bit processor with a three-stage instruction pipeline, an integer multiplier, 256 MB DDR3L memory, and a direct memory access (DMA) controller. Xilinx’s Vivado design suite (Xilinx, San Jose, CA 95124, USA) was used to develop and simulate the VHDL code for the correlator and the MicroBlaze microprocessor. It also was used to program the FPGA development board. Xilinx’s Vitis IDE was used to develop the software running on the MicroBlaze processor.

**Figure 11:**
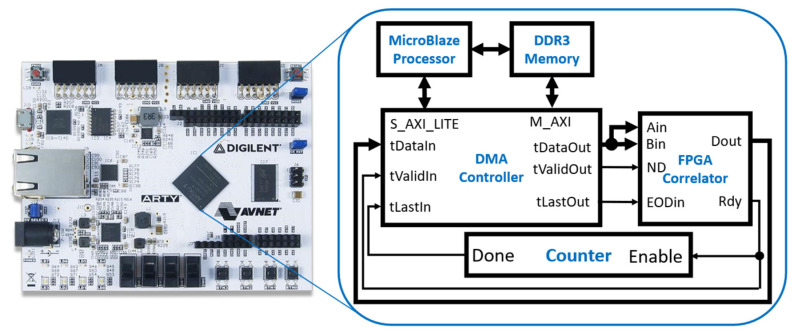
The test bench includes the MicroBlaze processor and the FPGA correlator, which can be configured with either the linear or multi-tau correlator. The DMA controller has a write channel to send data to the correlator and a read channel to receive the data from the correlator. The test bench was implemented on the Arty A7 board.

Figure 11 is the block diagram of the test bench design. The DMA controller is an AXI DMA module available in Vivado and has one write channel and one read channel. The write channel has the data port (tDataOut), data valid signal (tValidOut), and the last data indicator (tLastOut). They were connected directly to the data port (Ain and Bin), Nd, and EODin of the FPGA correlator, respectively. The outputs of the FPGA correlator were connected to the read channel of the DMA controller with the Dout connected to tDataIn and Rdy signal connected to tValidIn. The counter counts the Rdy pulses, and its output (Done) goes active high when the counter value reaches 80, the total number of output correlation values. The Done signal is connected to the tLastIn of the read channel. M_AXI is the data port of the DMA controller and S_AXI_LITE is the configuration port of the DMA controller.

The processor generated a test data set in memory and sent it to the correlator through the DMA write channel. The correlator then sent the computed correlation data back to the processor’s memory via the DMA read channel. An interrupt was generated when the tLastIn signal went active high to notify the microprocessor. The microprocessor and correlator were running at 100 MHz, and a global reset signal was used for all synchronous components.

Linear and multi-tau correlators were also created in software with C in Vitis. They used the same algorithms and single-precision data as the FPGA correlators and served as a baseline for comparison with the FPGA output. The software was run on the same MicroBlaze processor implemented on the Arty A7 FPGA development board. The same data sets were used on both the software and FPGA correlators. The outputs from both correlators were collected and compared. The mean square error (MSE) between the outputs of the FPGA correlator and the software correlator was calculated using the following equation:

(2) $$MSE=\frac{1}{80}\overset{79}{\underset{i=0}{\sum }}{\left(c_{f}\left(i\right)-c_{s}\left(i\right)\right)}^{2}$$

where $c_{f}$ is the output of correlation values from the FPGA correlator, $c_{s}$ is the output of correlation values from the software correlator, and 80 is the number of correlation values in the tests.

The performance of the FPGA correlator was evaluated by comparing its computation time with the software correlator running on the Xilinx MicroBlaze microprocessor. The computation time of the FPGA correlator was the difference between the time of the input of the last data point and the time of the output of the first correlation value. A counter was implemented in the FPGA correlator that started counting the FPGA clocks when the EODin input signal of the correlator went active high. This is the time of the last data entry to the correlator. The counter stopped counting when the Rdy output signal of the correlator went active high. This is the time of the output of the correlation values. The counter’s value is the latency of the FPGA correlator. The computation time of the software correlator was the time to complete the correlation on the same data set. A counter was added to the test bench to measure the execution time. It was started at the beginning of the software computation of the correlation and stopped at the end of the computation. The counter value was the number of processor clocks used in the software correlation computation. Both counter values were converted to time durations by multiplying them by 10 ns, the period of the 100 MHz working frequency.

## 3. Results

The tests were performed on both the linear and multi-tau correlators. Both correlators output 80 correlation lags as illustrated above. For convenience, the correlation lag was represented by integers as the unit lag time was equivalent to the data sampling interval. The absolution time of the correlation lag is not used in the correlation computation. Therefore, we used integers to represent the correlation lags with the value 1 as the unit lag. For the multi-tau correlator, the unit lag remained constant for the first 16 correlation lags and doubled every eight lags thereafter. Three sets of data were used in the tests: a constant data series, a pulse series, and a sine wave series. The results from the software correlator were used to validate the FPGA correlator. The autocorrelation was selected for these tests so that only one data series was used for the correlator inputs Ain and Bin. The data sets were selected so that their correlation values could also be validated by theoretic values.

### 3.1. Linear Correlator

**Figure 12:**
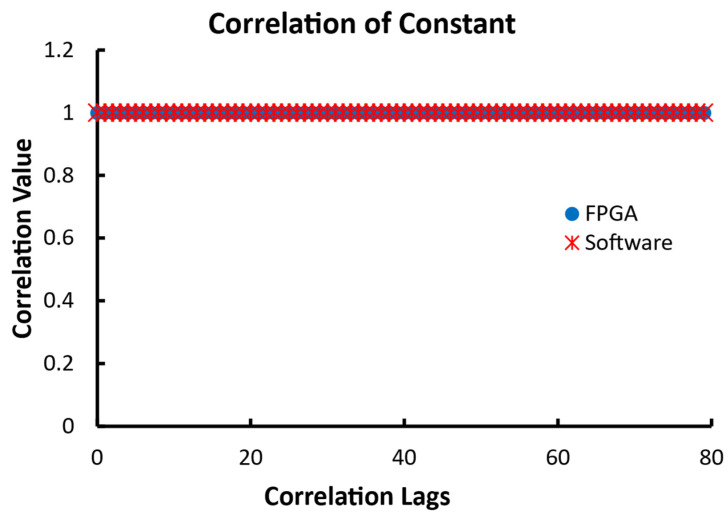
The outputs of 80 correlation lags from the linear FPGA correlator and the linear software correlator when the input data are constants of 1.

The size of the three data series was 1000 data points each. The first set was a series of constant values of 1. The results from this input for both the FPGA and software correlators are shown in Figure 12. Both the FPGA and software correlators output the identical result of 1 for all of the lags, as expected for the given input. That can be easily validated using Equation (1).

**Figure 13:**
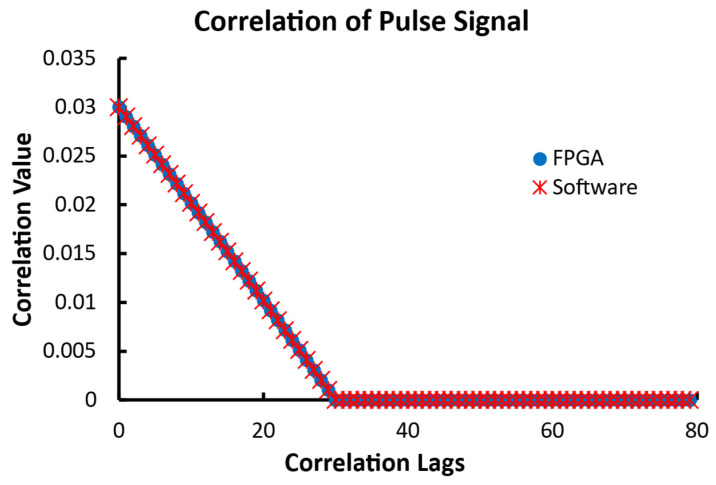
The outputs of 80 correlation lags of a pulse with 30 data points of value 1 followed by 970 zeros from the linear FPGA correlator and the software correlator.

The second data set for the linear configuration was a pulse with a value of 1 for the first 30 data points and 0 for the remaining 970 points. As shown in Figure 13, both the FPGA and software correlators output identical results for every lag. Expectedly, the autocorrelation has the largest value of 0.03 at the lag of 0 and decreases as the lag increases until 30. The correlation becomes 0 since the original and delayed pulses are no longer overlapped beyond that point of lag.

Mathematically, the input data series is defined by the following Equation (3):

(3) $$x\left(n\right)=\{\begin{array}{c} \;1\;\;\;when\;n<30\\ \;0\;\;\;when\;n\geq 30 \end{array}$$

c(n) is the correlation of x(n), and its values are computed from Equation (4) when n is less than 30. The correlation values are zero when n is greater than or equal to 30.

(4) $$c\left(n\right)=\frac{30-n}{1000-n}\;\;\;\;\;\;when\;n<30$$

Thus, the correlation at the zero-lag c(0) is 0.03 based on Equation (4).

The last tested 1000-point data set for the linear configuration was a sine signal. The signal had a period of 20 data points with a maximum value of 2047 and a minimum of −2047 and an initial phase of zero. The results for this input are shown in Figure 13. Again, the FPGA and software returned identical results. The expected result of the autocorrelation of a sine function is a cosine function with the same period.

Mathematically, the input data series is defined by the following Equation (5):

(5) $$x\left(n\right)=2047\sin\left(\frac{n\pi }{10}\right)$$

The theoretic values of the correlation are defined in Equation (6).

(6) $$c\left(n\right)=\frac{2047^{2}}{2}\cos\left(\frac{n\pi }{10}\right)$$

**Figure 14:**
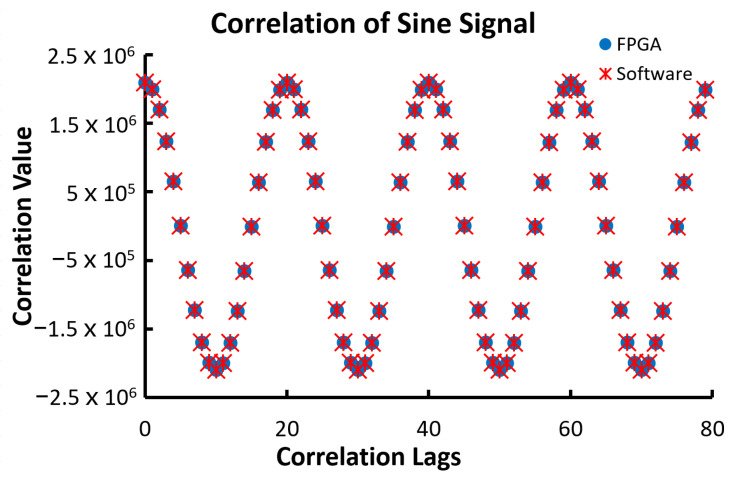
The outputs of 80 correlation lags of a sine signal with the amplitude of 2047 and the period of 20 data points from the FPGA correlator and the software correlator.

Therefore, the theoretic value of correlation at the zero-lag c(0) is 2.06 × 10^6^, and the correlation is a cosine function, as shown in Figure 14.

Table 2 is the MSE between the FPGA linear correlator and the software linear correlator. The MSE of zero demonstrated both outputs were identical.

### 3.2. Multi-Tau Correlation

**Figure 15:**
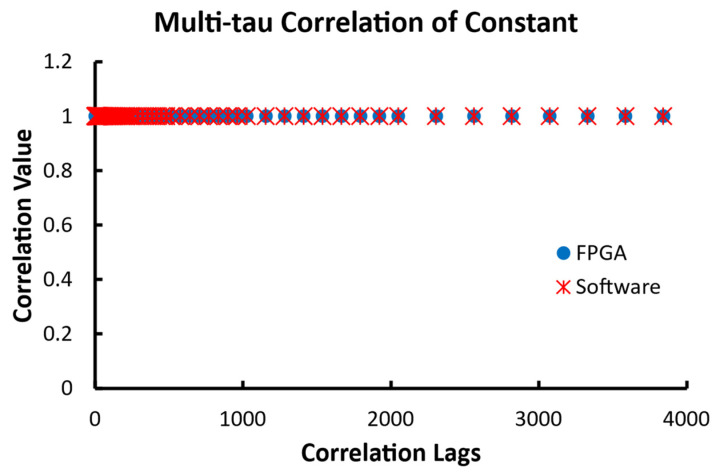
The outputs of 80 correlation lags from the multi-tau FPGA correlator and the multi-tau software correlator when the input data are constants of 1. The correlation lag doubles every 8 lags after the initial 16 lags.

The test of the multi-tau correlator used 10,000-point data sets for the multi-tau correlation of the constant signal and the pulse signal. Since the unit time lag is doubled every eight lags after the initial 16 correlation lags, the last lag from the correlator is at 3840 when the initial lag is used. The large data size allows for the calculation of the correlation at much larger lags. The first data set was a series of constant values of 1. The results from this input are shown in Figure 15. The output of the 80 correlation values in the figure shows the increase in the correlation lag. As expected, both the FPGA and software returned the identical result of 1 for all lags.

**Figure 16:**
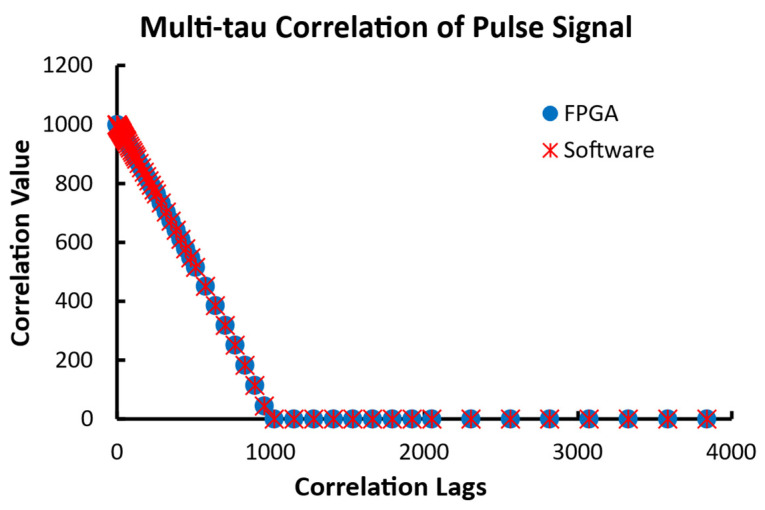
The outputs of 80 correlation lags of a pulse with 1000 data points of value 100 followed by 9000 zeros from the multi-tau FPGA correlator and the software correlator. The correlation lag doubles every 8 lags after the initial 16 lags.

The second data set was a pulse with a value of 100 for the first 1000 data points and 0 for the remaining 9000 points. As shown in Figure 15, both the FPGA and software implementations output identical results for every delay. Similar to the case in the linear correlator, the theoretic value of the correlation when n is less than 1000 is listed in Equation (7) and zeros for other correlation values. Therefore, the correlation value at zero-lag c(0) should be 1000, as shown in Figure 16.

(7) $$c\left(n\right)=\frac{100^{2}\left(1000-n\right)}{10,000-n}\;\;\;\;\;\;when\;n<1000$$

**Figure 17:**
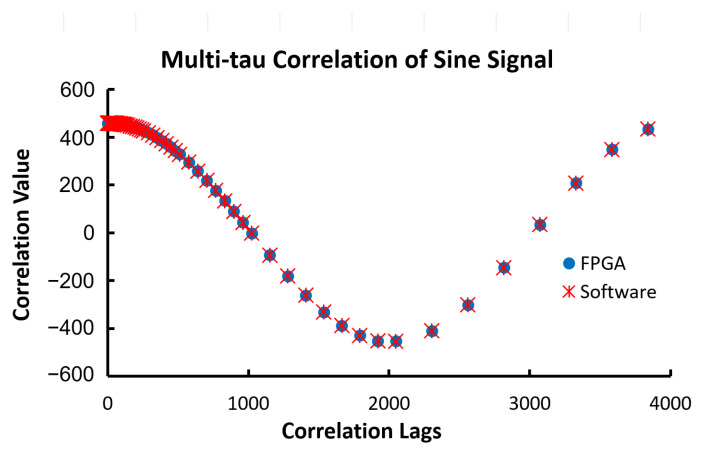
The outputs of 80 correlation lags of a sine signal with the amplitude of 31 and the period of 4000 data points from the multi-tau FPGA correlator and the software correlator. The correlation lag doubles every 8 lags after the initial 16 lags.

The third data set was a sine signal with a period of 4000 data points and a total size of 20,000 data points. This allows the correlator output to cover the entire cycle of the correlation. The larger data size reduces the windowing effect caused by the finite data size. The amplitude of the sine signal was 31 and the initial phase was zero. Thus, the correlation should be a cosine function with an amplitude of 480, as shown in Equation (6). Figure 17 shows the output from both the FPGA and software correlators; both outputs matched perfectly.

Table 3 is the MSE between the FPGA multi-tau correlator and the software multi-tau correlator. The MSE of zero demonstrated both outputs were identical.

### 3.3. Performance

The time between the entrance of the last data point into the correlator and the first data output is 140 ns for the linear correlator and 150 ns for the multi-tau correlator because of the additional scaler module. This latency is independent of the size of the data series. The computation time of the FPGA correlator and the software correlator running on the MicroBlaze processor is listed in Table 4. It is obvious that the FPGA correlator is at least 85,000 times faster than the software correlator running on the MicroBlaze processor. The computation time of the FPGA correlator did not change as the data size changed, but the software correlator showed that the execution time was proportional to the data size.

## 4. Discussion

All tests on the FPGA correlator designs yielded identical results to those on the software correlator. The results were also validated by the theoretic values of the correlation of the data sets. This indicated that the FPGA correlator produced an accurate correlation output and that the hardware design is correct. The benefits of the FPGA correlator over the software correlator are summarized in Table 5. The high efficiency of the FPGA correlator allows for the correlation computation to be offloaded to FPGA hardware and makes the combination of microcontrollers and FPGA attractive in a portable embedded device for real-time data processing.

The advantages of the design are simplicity and scalability. Compared to the work of Islambek et al., no state machine is needed to coordinate the operation of the FPGA correlator. Furthermore, the implementation of the multi-tau scheme does not require a set of clock signals for the data downsampling. The modular approach makes the design highly flexible. The basic building block of the correlator is the SLC module. It can be used to build linear or multi-tau correlators with any number of correlation lags. The connections of the modules are direct, and no additional circuitry is needed. The data flow between modules is regulated by the handshake signals: ND for the input data and Rdy for the output data. The design is compatible with the non-blocking mode of the Xilinx IP modules available through the Vivado suite, which simplifies the connections of FPGA modules in a pipelined design for parallel processing. The EOD signal indicates the last data point for the correlation. Although it is shown to be synchronized with the ND signal in Figure 2 and Figure 4, it can go active at any time to indicate that the data point validated by the latest ND signal is the last data point. That allows the combiner modules to work when the number of input data is odd. In that case, the combiner will drop the last data point by not activating its Rdy output. The EOD still works for the following modules even though it is delayed from the last ND signal.

Our design has applications in optical biosensing technologies such as DCS, DLS, and FCS. DCS and DLS require the autocorrelation of the photon counts of the scattered light induced by a coherent laser source. DCS can measure the blood perfusion in soft tissue. It derives the blood flow index from the autocorrelation of the scattered light. Detailed descriptions of the device can be found in the literature [25,26,27]. DLS is widely used in biosensing to study nanoparticles, proteins, and their interactions [28]; measure nanoparticle size [29]; and detect specific inhibition of nanoparticle aggregation [30].

FCS works slightly differently from DCS and DLS. It uses a confocal system to collect the fluorescence emitted from the fluorescently labeled molecules in the confocal area. The autocorrelation of the fluorescent light is computed as the initial data to investigate the mobility of the fluorescent particles and molecular interactions of proteins [31].

**Figure 18:**
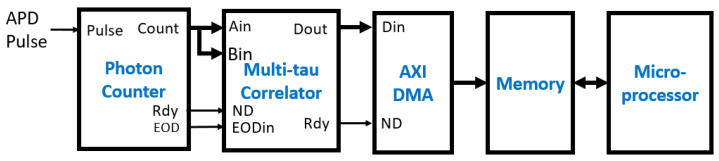
FPGA multi-tau correlator in an embedded diffuse correlation spectroscopy device using a microprocessor.

The FPGA correlator can be easily integrated into the DCS, DLS, and FCS by inserting it between the Avalanche Photon Detector (APD) and the microprocessor. Figure 18 is the illustration of the sensing and analysis component of an embedded DCS design using an FPGA multi-tau correlator with a microprocessor. The APD outputs the pulses of the detected photons, which are counted by the photon counter module within a preset time window, e.g., 200 ns or 5 Ms/s sampling rate. At the end of each time window, the photon counts are output at the count port and sent to the Ain and Bin ports of the multi-tau correlator for computation of the autocorrelation. At the same time, the end of the time window triggers the Rdy signal, which is connected to the ND input of the multi-tau correlator. The EOD pulse is generated when the preset number of data points is reached. The output data from the correlator are sent to memory through the AXI DMA module by Xilinx. The processor can then start processing the correlation values in the memory for the blood flow index. For example, 20 ms of photon count data have 100 k samples at the sampling rate of 5 Ms/s. If the FPGA correlator is used, the time to compute the correlation is less than 1 ms, which allows the processor to generate 50 blood flow index values per second. This is significant for the monitoring of dynamic blood flow in tissue between heartbeats because the signal duration is typically less than one second. However, if the software correlator is used, it will take at least 0.4 s to generate one blood flow index value for the same data size. This is because the computation time for software is proportional to the data size, as shown in Table 4, and 400 ms is needed to compute the autocorrelation of 100 k samples using the multi-tau scheme. Thus, the sample rate will be around 2 s/s for the software correlator, and it cannot provide the essential time resolution of the blood flow between heartbeats. The FPGA correlator enables the real-time monitoring of blood flow in tissue with an embedded DCS device powered by a microprocessor.

The nature of the multi-tau correlation scheme is related to signal sampling rate. When the number of correlation lags becomes large, the original sampling rate will be excessive. The increase in the time of the unit correlation lag is equivalent to the downsampling. There are two approaches for the multi-tau correlation. The first approach uses the average of two consecutive samples as the new sample value. That effectively downsamples the signal by two. However, the sample values could lose precision if they are small because they are integers and the average could lose one significant bit. For example, in DCS, the data are the photon counts of the received scattered light. The typical count is between 100 k and 700 k per second [32]. If the initial sample window is 100 ns, the count within that time frame is typically zero or one. Dividing a small, odd integer by two would result in a significant loss of precision, and it becomes worse as the multi-tau step progresses. The second approach addresses this deficiency by using the sum of the two consecutive samples, i.e., the total counts in a wider time window. That also introduces a factor of 4 in the final value for each combiner, which is compensated for by the scaler module at the end of the multi-tau correlator. However, it limits the maximum value of the data. That was the reason we chose 31 as the amplitude of the sine signal in the test of the multi-tau correlator. It is less of a concern in optical applications, such as diffuse correlation spectroscopy, because the 16-bit data width provides a sufficient cushion for the value expansion by the combiner module.

The use of floating-point data to normalize the correlation values to the number of data points in the correlation can keep the precision of the correlation values and make the following processing in software easy. This feature was not available in the published literature. Although a single-precision floating-point format is used in our design, a double-precision floating-point format can also be used. It is a simple change in configuration of the Xilinx floating-point IP, though it comes at the cost of using more DSP slices and logic slices in the FPGA. This is a balance between FPGA resource utilization and convenience in software processing.

The FPGA correlator also has the following limitations: First, overflow is a major risk in the FPGA correlator, and its detection is not available in the current design. Mitigation of overflow in the design is accomplished by selecting a sufficient width of the input data and the accumulator in the MAC. These data widths are customizable and limited by the DSP slices in the FPGA. The input data width can be as high as 18 bits and the accumulator width can be as high as 48 bits, which gives much more room for the accumulation to grow. Second, since the design is intended for universal use, it does not consider the power consumption and the utilization of fabric in the FPGA. Such issues should be resolved in a real application by choosing the right FPGA chip of sufficiently low power consumption and adjusting optimization settings for the efficient use of FPGA fabric. Lastly, the correlator was not tested using biosignals in the current study. Our future work is to build the embedded DCS illustrated in Figure 18. It will be clinically significant to test and validate the device using the biosignals from in vivo experiments.

In summary, the design of the FPGA correlator in this paper presented a simple hardware solution for correlation computation. It can be the hardware accelerator of a microcontroller for applications requiring high computational power for real-time correlation analysis. It can also take advantage of system-on-chip technology, such as the Xilinx Zynq SoC chip, to create device-on-chip solutions with the integration of sensors. The FPGA correlator is one of the modules that can power these device-on-chip applications.

## Figures and Tables

**Table 1:** Signal definitions of the correlator module.

Signal	Description
Ain	The first input data series in the format of a 16-bit signed integer.
Bin	The second input data series in the format of a 16-bit signed integer.
ND	Active high signal to indicate valid data at Ain and Bin.
EODin	Active high signal indicating the input of the last data point.
Aout	Direct output of Ain.
Bout	Delayed output of Bin based on the ND signal.
Rdy	Active high signal to indicate valid data at Aout and Bout.
EODout	Direct output of EODin.
Dout	The output of the raw correlation value in the format of a 32-bit signed integer.
Nout	The output of the number of data points used in the calculation of the raw correlation value at the output Dout. It is in the format of a 16-bit signed integer.
DRdy	Active high signal to indicate valid data at Dout and Nout.
Din	The input of the raw correlation values from the following cascaded correlation module in the format of a 32-bit signed integer.
Nin	The input of the number of data points used to calculate the raw correlation value at the input Din. It is in the format of a 16-bit signed integer.
DND	Active high signal to indicate valid data at Din and Nin.
Reset	Active high signal to reset the registers in the module (not shown in figures).
Clock	FPGA working clock (not shown in figures).

**Table 2:** MSE of the linear correlator.

Signal Type	MSE
Constant	0
Pulse	0
Sine	0

**Table 3:** MSE of the multi-tau correlator.

Signal Type	MSE
Constant	0
Pulse	0
Sine	0

**Table 4:** Computation times of the FPGA and software correlators on the same sets of data.

Correlator Type	Signal Type	Data Size	Time (FPGA)	Time (Software)	FPGA:Software
Linear	Constant	1000	140 ns	12.11 ms	1:86,500
	Pulse	1000	140 ns	11.91 ms	1:85,070
	Sine	1000	140 ns	12.11 ms	1:86,500
Multi-tau	Constant	10,000	150 ns	39.96 ms	1:266,400
	Pulse	10,000	150 ns	39.85 ms	1:265,700
	Sine	20,000	150 ns	78.93 ms	1:526,200

**Table 5:** Comparison of the FPGA correlator with the software correlator.

FPGA Correlator	Software Correlator
Parallel data processing	Serial data processing
Computation time is independent of data size.	Computation time is dependent on the data size.
Computation can be finished within one microsecond.	Computation can be finished over dozens of milliseconds.

## Data Availability

The data in this study are not publicly available but may be obtained from the corresponding author upon reasonable request. The VHDL code for the correlator modules is available on GitHub on 5 April 2022 (https://github.com/chmoore889/FPGA-Correlator/tree/master/src/design).
